# Xylitol and Maltitol Improve the Rheological Property of Kappa-Carrageenan

**DOI:** 10.3390/foods11010051

**Published:** 2021-12-27

**Authors:** Min Huang, Yihan Mao, Yuzhu Mao, Hongshun Yang

**Affiliations:** 1 School of Food Science and Biotechnology, Zhejiang Gongshang University, Hangzhou 310018, China; huangmin@zjgsu.edu.cn; 2 Department of Food Science and Technology, National University of Singapore, Singapore 117542, Singapore; e0427625@u.nus.edu (Y.M.); e0427545@u.nus.edu (Y.M.); 3 Center for Peak of Excellence on Biological Science and Food Engineering, National University of Singapore (Suzhou) Research Institute, 377 Lin Quan Street, Suzhou Industrial Park, Suzhou 215123, China

**Keywords:** carrageenan, polyol, rheology, thermoreversibility, Fourier transform infrared (FTIR) spectroscopy, polysaccharide

## Abstract

To further extend the use of κ-carrageenan (κ-C) in real food systems (such as beverages), the understanding of gelation properties of κ-C with the presence of food ingredients is critical. The effects of xylitol and maltitol (up to 30 wt %) on the rheological and structural properties of κ-C were inspected by means of rheometer and Fourier transform infrared (FTIR). With the addition of xylitol, the gelation temperature increased from 44.1 to 57.3 °C, while the gelation temperature increased from 44.1 to 61.4 °C in maltitol systems. With the increasing concentration of both xylitol and maltitol, the values of fractal dimension *d_f_* and complex modulus G* of κ-C increased, while the relaxation exponent *n* decreased from 0.87 to 0.39 of xylitol and 0.87 to 0.78 of maltitol, respectively. These indicated that the gel networks of aqueous κ-C were improved by the addition of xylitol and maltitol. The FTIR results showed that the interaction between κ-C and these polyols contributed to the increase of hydrogen bonds. The effects of maltitol on κ-C were stronger than those of xylitol because of more equatorial-OH bonds in maltitol. These findings contribute to a better understanding of the gelation processes of κ-C/polyols systems.

## 1. Introduction

Carrageenan is a series of linear sulfated polysaccharides extracted from red edible seaweeds. They have been extensively used in food industry because of their gelation, thickening, and stability [1]. Among the three main types (kappa, iota and lambda-carrageenan), kappa-carrageenan (κ-C) has attracted extensive interest because of its high gel forming ability, transparency, and thickening property [2].

The gel formed by κ-C has thermoreversibility, which means that the gel melts into a solution upon heating and forms a gel when the solution is cooled [3]. As a consequence, in the food industry, κ-C is an excellent coagulant for making fruit jelly. It can be solidified at room temperature with good stability and good heat transfer [4]. Furthermore, κ-C can also be used in aquatic products, and meat products such as sausage and ham meat [5].

In earlier study, pure κ-carrageenan has been investigated and concluded that the gelling process of κ-C included the transition from coil to helix and the double helices aggregation [6,7]. The gelation of κ-C could be impacted by the temperature, some positive ions, and the addition of co-solute, sugars and polyols. Among these factors, the addition of polyols in κ-C system is one important method to improve the gelling properties of κ-C in food processing. Indeed, there had been considerable attention paid to the effects of sugar and polyols addition on κ-C gel. With increasing amount of the sucrose and polysaccharide, the gel network was ameliorated and the water-holding capacity of the gel was improved [8,9]. The influences of sugars and polyols on the transition process of κ-C from sol to gel were examined by differential scanning calorimetry (DSC). The addition of these compounds always rose in the gelling temperature, and when its concentration increased, there was a decrease enthalpy of gelation, which might result from polymer-solvent hydrogen bonding replaced by polymer-polymer hydrogen bonding [10]. However, how polyols actually strengthen the gelation in terms of rheological properties and microstructure as affected by κ-C levels still needs further study.

Xylitol is categorised as a 5-carbon polyalcohol or sugar alcohol (specifically an alditol) [11]. Maltitol, also called 4-O-α-d-glucitol, is a carbohydrate and a sugar alcohol [12]. Xylitol and maltitol have many excellent physicochemical properties, such as stable nature, certain sweetness, hygroscopicity, and emulsion stability [13]. These two polyols have been widely used in food, chemical, and cosmetic industries. What’s more, xylitol and maltitol were used as functional sweeteners along with κ-carrageenan as the thickener to produce the low sugar yogurt [14,15]. Thus, it is essential to study how xylitol and maltitol influence the gel properties of κ-carrageenan.

This study aimed to study the effects of different concentrations of xylitol and maltitol on κ-C. The thermoreversible behaviour, critical viscoelastic behaviour, small deformation rheological characteristics of κ-C with different xylitol and maltitol concentrations were measured by rheometer and analysed. The effects of different concentrations of xylitol and maltitol on the microstructure of κ-C were also measured by FTIR and elucidated. Finally, a schematic mechanism was proposed to expound all the results. This study adds our knowledges about the κ-C behaviour in the presence of polyols such as xylitol and maltitol.

## 2. Materials and Methods

### 2.1. Materials and Sample Preparation

κ-Carrageenan (κ-C, TCI, Tokyo, Japan) at 2 wt % concentration was mixed with xylitol and maltitol (TCI, Japan) to make 0, 10, 20, 30 wt % solutions using Millie-Q water and heated in a water bath (80 °C) with mild stirring for 120 min. The powder was completely swollen to form homogeneous solutions as shown in Figure S1.

### 2.2. Rheological Measurements

The rheological measurements were carried out on a rotational rheometer (DHR, TA Instruments, New Castle, DE, USA) with a plate-plate geometry possessing diameter of 40 mm with a gap of 1.0 mm in clearance and a Peltier temperature control. Sample in capped glass vial was transferred to the bottom plate of the rheometer directly using pipette. The bottom plate was preheated to 80 °C and then the sample was loaded at 80 °C for 10 min to melt all the probable helical structures [16]. A layer of sunflower oil around the perimeter of parallel plate was dropped to prevent water evaporation. All tests were carried out in triplicates.

#### 2.2.1. Strain Sweep Measurement

The range of strain sweep measurement was 0.1–100% and the angular frequency was 1 rad/s to obtain the linear viscoelastic region (LVR) of the sample in solution and gel state [17].

#### 2.2.2. Temperature Sweep Measurement

Temperature sweep measurement was performed from 80 °C to 20 °C to acquire the sol-gel transition temperature. Then the sample was kept at 20 °C for 5 min. After that, the measurement was carried out from 20 °C to 80 °C to acquire the melting temperature. The cooling and heating rates were 2 °C /min, the angular frequency was 1 rad/s, and the fixed strain was 2% (in the LVR region based on the results of Section 3.2).

#### 2.2.3. Frequency Sweep Measurement

Firstly, the sample was held at 20 °C for 3 min for soaking. The frequency sweep (1–100 rad/s) measurements were conducted at 5 constant temperatures near the gelation temperature obtained in the temperature sweep measurement at a fixed strain of 2% [18].

Traditionally, most studies used the crossover between *G*′(ω) and *G*″(ω) to serve as indicators of gel sites. However, this means is ineffective since gelling point is affected by the cooling rate and frequency. Chambon and Winter [19] discovered the scaling law of *G*′ (ω) = *G*″ (ω) ~ ω^1/2^ in the gel point experiment including chemical and physical gels. The storage modulus *G*′ and the loss modulus *G*″ can be demonstrated:

*G*′(ω) ~ *G*″(ω) ~ ω*^n^* (1)

In addition,

(2) $$\tan\;\delta =\frac{G{}^{\prime \prime }\left(\omega \right)}{G{}^{\prime }\left(\omega \right)}=\tan\left(\frac{n\pi }{2}\right)$$

where tan *δ* is the loss factor, and *n* means critical relaxation exponent, respectively.

Assuming that the hydrodynamics and volume exclusion interactions can be included and entanglement effects can be ignored, the relationship between the relaxation exponent *n* and the fractal dimension *d_f_* can be expressed as:

(3) $$n=\frac{d\left(d+2-2d_{f}\right)}{2\left(d+2-d_{f}\right)}$$

where *d* (*d* = 3) indicates the space dimension, and *d_f_* means the fractal dimension, respectively.

#### 2.2.4. Small Deformation Oscillatory Shear Rheology

Time sweep measurement was performed at following conditions: temperature change rate of 2 °C/min, 1 rad/s angular frequency, 2% strain, and 10 min soak time to form the gels. Then the gel network with the time sweep measurement was formed at 20 °C for 300 min at an angular frequency of 1 rad/s and a strain of 2%. Finally, the frequency sweep measurement (0.1–100 rad/s) was undertaken at 20 °C with a constant strain 2% [8]. To better compare the implications of xylitol and maltitol on the gel network of κ-carrageenan, the complex modulus G* was conducted, as follows:

(4) $$G^{*}={\left({G{}^{\prime }}^{2}+G{{}^{\prime \prime }}^{2}\right)}^{0.5}$$

### 2.3. Fourier Transform Infrared (FTIR) Spectroscopy Measurement

All samples were freeze-dried and manually ground with KBr powder (Merck KGaA, Damstadt, Germany) at a same ratio (sample:KBr = 3:97). The KBr powder was stored at 100 °C for at least 12 h before using to eliminate any moisture absorbed. Then the samples were grinded and baked under infrared heat lamp. After that, the press machine (1 t) was conducted on the powder. The pellets were then tested using a Spectrum One FTIR spectrometer (PerkinElmer, Waltham, MA, USA). Background spectrum was collected before each scan and spectra were collected over the range of 4000 and 450 cm^−1^ with a resolution of 4 cm^−1^ and 32 scans [20,21].

### 2.4. Statistical Analysis

Each test was conducted independently at least three times and the results are expressed as the mean ± the standard deviation (SD). Statistical analysis was performed using ANOVA followed by a Student-Newman-Keuls (SNK) procedure as implemented in the SPSS (IBM Corp., Armonk, NY, USA). Differences with a *p* value < 0.05 were considered statistically different.

## 3. Results

### 3.1. Effects of Xylitol and Maltitol on Thermoreversible Behaviour of κ-C

**Figure 1:**
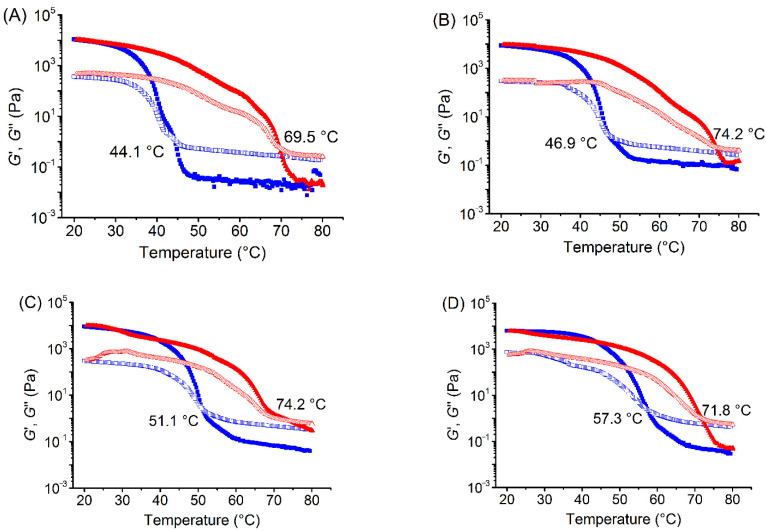

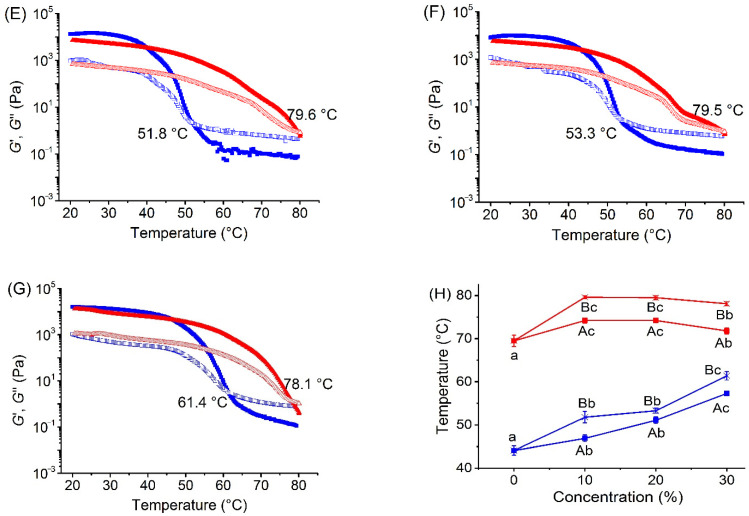
Storage modulus (*G*′) and loss modulus (*G*″) during temperature sweep for 2 wt % κ-carrageenan (κ-C) with the addition of (**A**) 0 wt % xylitol and maltitol; (**B**) 10 wt % xylitol addition; (**C**) 20 wt % xylitol addition; (**D**) 30 wt % xylitol addition; (**E**) 10 wt % maltitol addition; (**F**) 20 wt % maltitol addition; (**G**) 30 wt % maltitol addition (■: *G*′-cooling; **□**: *G*″-cooling; ▲: *G*′-melting; △: *G*″-melting). (**H**) The transition temperature of different samples (red line: melting temperature (*T*_m_); blue line: gel temperature (*T*_g_); square means the addition of xylitol; cross symbol means the addition of maltitol; different lowercase letters mean significant difference among different concentration, and different uppercase letters mean significant difference between xylitol and maltitol addition).

Figure 1 gives the information about storage modulus (*G*′) and loss modulus (*G*″) during the cooling and heating process of pure κ-C, κ-C/xylitol mixtures, and κ-C/maltitol mixtures. In the cooling process for all samples, *G*′ was less than *G*″ at the beginning, and both of them increased gradually as the temperature decreased. When a certain temperature was reached, both *G*′ and *G*″ grew rapidly, and *G*′ increased at a rate higher than that of *G*″, suggesting that the elastic component of the system began to increase. The system began to transform into gel until *G*′ and *G*″ intersecting, at which point the junction temperature was the gel temperature (*T*_g_). Therefore, the certain point at which *G*′ = *G*″ or tan *δ* =1 could be identified as the sol-gel temperature [22]. As the temperature declined further, *G*′ decreased faster than *G*″, indicating that the aggregates dissociated and the gel network was destroyed. This was because carrageenan molecules first formed a single-helical structure from the irregular clutter structure as the temperature decreases. Then it began to form a double helix as the temperature further decreased. With the decreasing temperature, the double helix further associated with the aggregation and finally formed a complete ordered network structure. Hence, the following state of the sample was the gel state.

In the heating and melting process for all samples, the values of *G*′ were higher than *G*″ as temperature increasing. When *G*′ and *G*″ intersected at a certain point, it arrived at the melting temperature (*T*_m_). As the temperature increased further, *G*″ exceeded *G*′, which indicated that the system was converted into viscoelastic liquid. This might be caused by the loss of the double helix structure [23]. For all samples, the gel temperatures were always lower than the melting temperatures during the whole cooling and heating process.

The value of *T*_g_ for pure κ-C was 44.1 °C (Figure 1A), which was a little higher than the reported temperature 39.4 °C by Wang et al. [24]. This might be due to the difference of κ-C source and purity. The *T*_g_ and *T*_m_ values of samples with xylitol and maltitol addition increased when compared with those of pure κ-C, and these temperature values became higher as the polyols concentration increased. Previous research reported the enhancement of κ-C sol-gel transition by sugars and polyols. Both xylitol and maltitol could increase the *T*_g_ and *T*_m_ values of κ-C [10], which was consistent with the phenomenon in this study.

The maltitol had a more remarkable effect on *T*_g_ and *T*_m_ values than xylitol at the same concentration. The equatorial-OH groups on the polyols were significant molecular characteristics, which could dominate the κ-C/polyols interactions in the sol state and were favourable for hydration [25]. In addition, the quantity of equatorial-OH groups in maltitol is more than that of xylitol based on their molecules. Thus, maltitol showed a more significant effect on *T*_g_ and *T*_m_ values of κ-C than xylitol.

The *T*_g_ values increased as the polyol concentration increasing. However, the *T*_m_ values showed no significant differences at 10% and 20% polyol concentration and had a significant decrease when the polyol concentration reached 30%. The possible reason might be that an increase in the concentration of polyols in the solution contributed to an increase in the initial temperature of the glass transition zone [26]. At higher polyol concentrations, aqueous polyols systems could form glasses with very different physical properties and correspondingly showed different effects on κ-C gels [27].

### 3.2. Effects of Xylitol and Maltitol on Critical Viscoelastic Behaviour of κ-C

**Figure 2:**
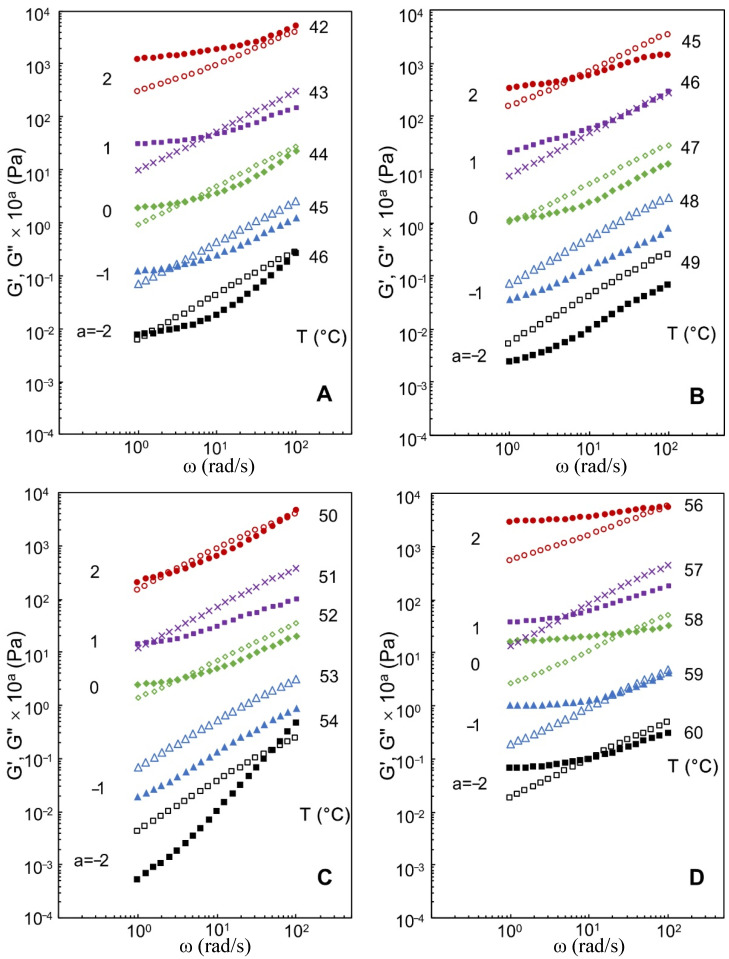

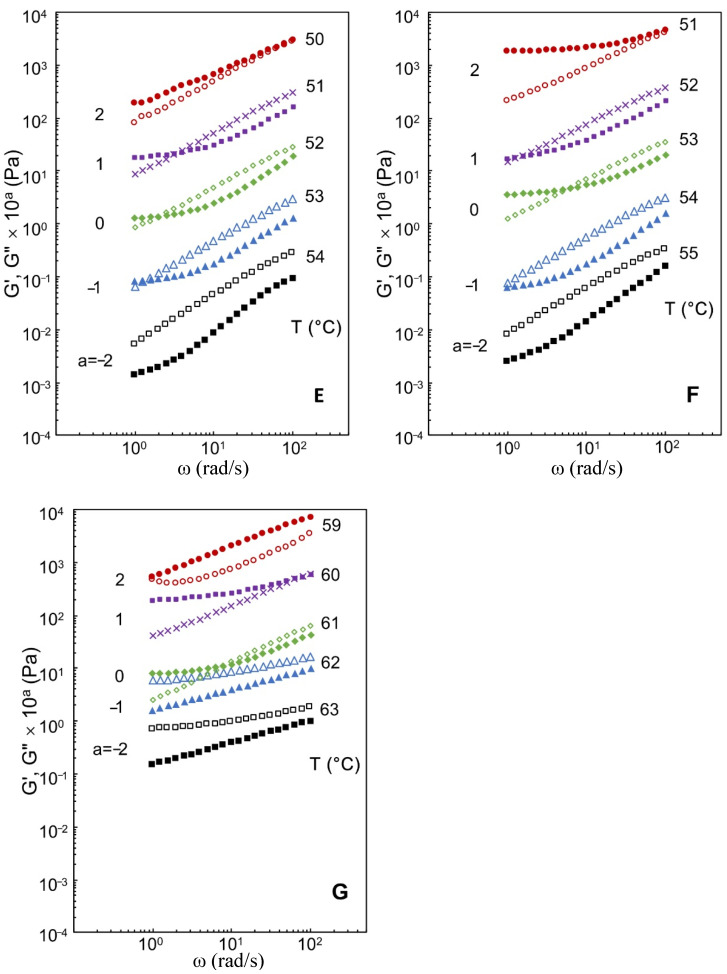
The values of *G*′ (solid symbols including ■, π, ♦, ■, and ●) and *G*″ (open symbols including □, △, ◇, ×, and ○) at different temperatures for 2 wt % κ-carrageenan (κ-C) with the addition of (**A**) 0 wt % xylitol and maltitol; (**B**) 10 wt % xylitol addition; (**C**) 20 wt % xylitol addition; (**D**) 30 wt % xylitol addition; (**E**) 10 wt % maltitol addition; (**F**) 20 wt % maltitol addition; and (**G**) 30 wt % maltitol addition. The values in figures indicated the data were calculated to move by a factor of 10^a^ vertically.

To investigate the gel system, it is essential to comprehend the determination of the gel point. Figure 2 illustrates the values of storage modulus *G*′ and loss modulus *G*″ for the xylitol and maltitol addition at five constant temperatures near the gelation temperature. At the high temperatures beyond the gel point, *G*″ was over *G*′ in the whole angular frequency range, indicating that typical viscoelastic fluid characteristics exceeded the gel point. With reduction of temperature *G*′ began to increase and was greater than *G*″. In the end, *G*′ platform appeared in the low-frequency region.

The loss factor and the relaxation exponent *n* were determined according to the gelation temperature and showed in Table 1. The value of *n* was in the range of 0~1 because of the phase angle δ varying from 0 to π/2. The decrease of *n* as polyols increasing indicated that the solution was changing from liquid to gel. This implied that the presence of hydrogen-bonded complexes in xylitol and maltitol could promote the coil-helix transition and helix aggregation of κ-C, and the formation of κ-C chain aggregates were occurred with higher concentration of polyols. It was in agreement with the results achieved by previous research [28].

The fractal dimension *d_f_* is depicted in Table 1. The values of *d_f_* increased as the concentrations of xylitol and maltitol increased. A greater value of *d_f_* resulted from a complicated structure [29]. Hence, the addition of xylitol and maltitol was benefit for the gel network. Similarly, this was because the helix aggregation.

Furthermore, the effects of maltitol on κ-C were stronger than those of xylitol in gel network, it might be resulted from the different amounts of OH-group in these two polyols. The polyols enhanced the hydrogen bonding network of the surrounding water molecules and also enhanced the hydrogen bonding network around the hydrophobic solute, thereby promoted the hydration enhancement which also correlated with the polyols-enhanced thermoreversibility of κ-C gel [25]. Thus, maltitol had a stronger effect on the gel properties of κ-C.

### 3.3. Effects of Xylitol and Maltitol on Small and Large Deformation Rheological Characteristics of κ-C

The sweep measurements in the previous process were the section of sol-gel transition. To explore the viscoelastic behaviour of κ-C with polyols addition, the small oscillatory deformation study was performed after the gel formed for 5 h. Table 1 presents the values of G* of different samples. As the concentration of xylitol and maltitol increased, the values of G* increased, which indicated that both xylitol and maltitol addition could promote the transition process from sol to gel and further aggregate the helices of κ-C, as a result they promoted the strength of gel network. It was also clear that the effect of maltitol was more obvious than that of xylitol at the same concentration. This might be because the density of hydroxyl groups available for intermolecular hydrogen bonding between maltitol molecules was greater than that of xylitol [30], which was consistent with previous results in this study.

### 3.4. Effect of Xylitol and Maltitol on the Structures of κ-C

**Figure 3:**
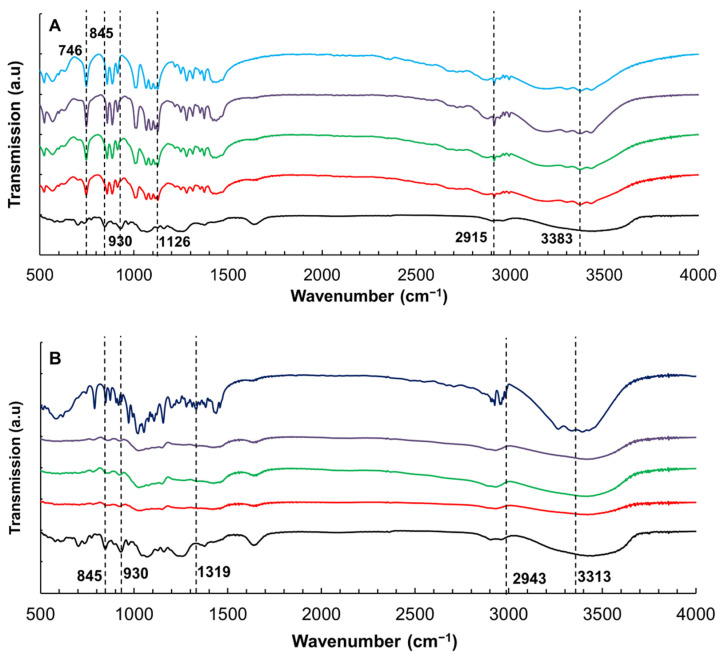
FTIR spectrum of κ-carrageenan (κ-C) with addition of xylitol (**A**) and maltitol (**B**). Pure xylitol and maltitol is also included as a reference. Black line: pure κ-C; red line: 10 wt % addition; green line: 20 wt % addition; purple line: 30 wt % addition; teal line: xylitol; blue line: maltitol.

Figure 3A reveals the FTIR spectra of κ-C with various amounts of xylitol. The pure κ-C and pure xylitol were included as control samples. The strong signals at 845 and 930 cm^−1^ were the characteristic peaks for κ-C [31,32,33]. These peaks can be observed in the κ-C spectrum. In samples containing xylitol, these two bands could not be observed, indicating these groups of κ-C disappeared through binding with xylitol. Furthermore, several large sharp peaks were observed and the FTIR spectrum of xylitol uncovered obvious vibrations at 746, 1126, 2915, and 3383 cm^−1^, which were consistent with previous reported spectrum of xylitol [34,35,36,37,38]. The signal of these peaks became weaker but still existed in the κ-C/xylitol mixtures, indicating that the combination between xylitol and κ-C did not change the structure of xylitol.

In the spectra of maltitol samples (Figure 3B), the representative peaks (at 845 cm^−1^ and 930 cm^−1^) of κ-C were also disappeared. According to the spectra of pure maltitol, the peaks exhibiting at 1319 and 2943 cm^−1^ were denoted by the C-H bonding [39]. The vibrational bands of free hydroxyl groups at 3313 cm^−1^ disappeared in all κ-C/maltitol samples, which could prove the formation of cross-linked hydrogen bonds in maltitol-κ-C molecules [27].

Furthermore, the effects of maltitol on κ-C were stronger than those of xylitol when compared the FTIR spectra (Figure 3). The FTIR absorption peaks of the κ-C/maltitol samples were quite different with that of both pure κ-C and pure maltitol, while the spectrum of the κ-C/xylitol samples were similar to that of the pure xylitol. This phenomenon indicated that more interactions were occurred in κ-C/maltitol systems. There are many factors that could account for the results. Firstly, the OH-group played a crucial role in molecular characterization by controlling the interaction between the solvent and the κ-carrageenan through the preferential hydration and the intermolecular hydrogen bonding [40]. The molecules of maltitol contained more OH-group than that of xylitol, resulting in more significant effect on κ-C. Secondly, there were polymers bound water in the mixture of κ-C and maltitol, which led to a more intense hydration.

### 3.5. Schematic Mechanism Analysis

**Figure 4:**
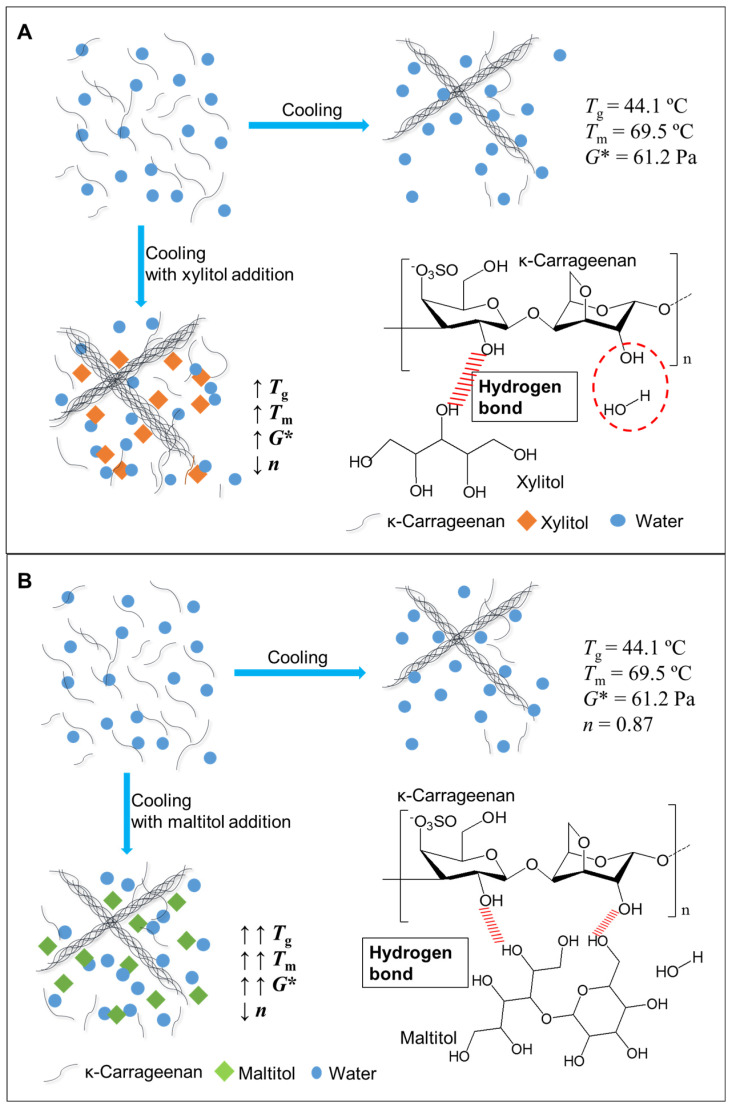
Schematic illustrations of the transition of κ-carrageenan (κ-C) gels as impacted by xylitol (**A**) and maltitol (**B**).

According to the previous results, a schematic illustration for the correlation of maltitol and xylitol with the gelation of κ-C was proposed and shown in Figure 4. The process of κ-carrageenan gelation generally involved in two steps: the transformation from coil to helix in junction zones, and the aggregation of double helix followed by gel formation at appropriate polymer concentration [41]. These coils can only be recovered if the aggregate melts at a higher temperature [42]. Polyols added to the κ-C solution had good hydration in the aqueous phase. The addition of polyols reduced the average water molecules around the κ-C chain [27]. Thus, the κ-C-water interaction was weakened and the κ-C chain was converted from self-binding to binding to the adjacent chain [24]. With the increase of polyols concentration, more bonds and double helix aggregates were formed in the transformation of helix structure. In addition, the helical combination of polyols and κ-C promoted the aggregation of cyclic junctions and formed larger junctions by forming intermolecular cross-linked bonds between κ-C and polyols [43]. Furthermore, thanks to the more amount equatorial-OH bonds in maltitol, there might be more hydrogen bonds between maltitol and κ-C than that of xylitol and κ-C. Therefore, the κ-C gel network had been enhanced more significantly.

## 4. Conclusions

This study systematically investigated the effects of xylitol and maltitol on the thermoreversible, critical viscoelastic, and structural properties of κ-C (2 wt %). The gelation temperature of κ-C increased from 44.1 to 57.3 °C after 30 wt % xylitol addition, while the gelation temperature increased from 44.1 to 61.4 °C with 30 wt % maltitol addition. As the polyol concentration increased, the values of the fractal dimension *d_f_* and the complex modulus G* increased while the relaxation exponent *n* values decreased, which indicated the gel networks of aqueous κ-C were improved by the addition of xylitol and maltitol. The FTIR results illustrated that the mixture of κ-C and polyols led to the formation of hydrogen bonds. A schematic mechanism was proposed to explain the enhancement of xylitol and maltitol on the coil-helix transition of κ-C. The addition of polyols reduced the average water molecules around the κ-C chain and then enhanced the gelation temperature. These findings can improve our understanding of the gelation process of κ-C/polyols systems, leading to the development of food product containing κ-C.

## Figures and Tables

**Table 1:** Dependence of the critical relaxation exponent *n*, fractal dimension *d*_f_, and complex modulus at 1 rad/s for κ-carrageenan gel with different xylitol and maltitol concentrations.

Samples	Critical Relaxation Exponent *n*	Fractal Dimension *d*_f_	Complex Modulus G* (Pa)
Control	0.87 ± 0.02 ^f^	2.30 ± 0.01 ^a^	61.2 ± 10.5 ^a^
10% xylitol	0.51 ± 0.01 ^c^	2.40 ± 0.02 ^b^	192.3 ± 57.1 ^b^
20% xylitol	0.48 ± 0.01 ^b^	2.40 ± 0.01 ^b^	229.7 ± 17.7 ^b,c^
30% xylitol	0.35 ± 0.01 ^a^	2.43 ± 0.01 ^c^	289.4 ± 30.2 ^c^
10% maltitol	0.82 ± 0.02 ^e^	2.31 ± 0.01 ^a^	251.2 ± 6.7 ^b,c^
20% maltitol	0.79 ± 0.01 ^d^	2.32 ± 0.01 ^a^	312.5 ± 17.7 ^c^
30% maltitol	0.78 ± 0.01 ^d^	2.32 ± 0.01 ^a^	411.5 ± 67.0 ^d^

Values are mean with standard deviation. Values with different superscripts in the same column are significantly different among different samples (*p* < 0.05).

## Data Availability

Data are available upon request.

## Supplementary Materials

The following are available online at https://www.mdpi.com/article/10.3390/foods11010051/s1, Figure S1: Pictures of analyzed gels. Samples from left to right are 2 wt % κ-carrageenan (κ-C) with the addition of 0 wt % xylitol and maltitol, 10 wt % xylitol addition, 20 wt % xylitol addition, 30 wt % xylitol addition, 10 wt % maltitol addition, 20 wt % maltitol addition, and 30 wt % malt-itol addition.
