# Supplementary material for: Xylitol and Maltitol Improve the Rheological Property of Kappa-Carrageenan
Source: Foods. 2021 Dec 27;11(1):51. doi: 10.3390/foods11010051 (PMC8750924; doi:10.3390/foods11010051)
Supplement: Supplementary file 1 [file foods-11-00051-s001.zip › foods-1498246-supplementary.pdf]

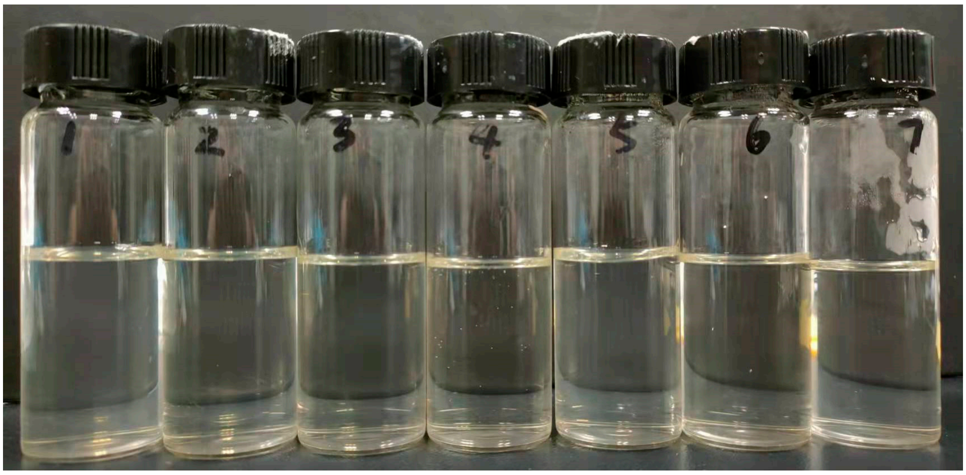

Figure S1. Pictures of analyzed gels. Samples from left to right are 2 wt% κ-carrageenan (κ-C) with the addition of 0 wt% xylitol and maltitol, 10 wt% xylitol addition, 20 wt% xylitol addition, 30 wt% xylitol addition, 10 wt% maltitol addition, 20 wt% maltitol addition, and 30 wt% malt-itol addition.
